# Ahmed Glaucoma Valves versus EX-PRESS Devices in Glaucoma Secondary to Silicone Oil Emulsification

**DOI:** 10.1155/2018/8539689

**Published:** 2018-06-20

**Authors:** Zhuyun Qian, Kai Xu, Xiangmei Kong, Huan Xu

**Affiliations:** ^1^Department of Ophthalmology and Visual Science, Eye, Ear, Nose, and Throat Hospital, Shanghai Medical College, Fudan University, Shanghai, China; ^2^Key Laboratory of Myopia, Ministry of Health, Fudan University, Shanghai, China; ^3^Shanghai Key Laboratory of Visual Impairment and Restoration, Fudan University, Shanghai, China

## Abstract

**Objective:**

To evaluate and compare the clinical effects of Ahmed glaucoma valves (AGVs) and EX-PRESS implants on glaucoma secondary to silicone oil (SO) emulsification.

**Methods:**

A retrospective case-series study was designed. A total of 23 eyes with late intraocular pressure (IOP) elevation secondary to SO emulsification were included in the study. Antiglaucoma surgery with implantation of AGVs or EX-PRESS devices was performed. Pre- and postoperative ocular parameters were recorded at each visit during a 1-year follow-up period. The rates of complete success (IOP < 21 mmHg without medication) and qualified success (IOP < 21 mmHg with ≤3 glaucoma medications) were analyzed.

**Results:**

A total of 14 eyes underwent AGV implantation, and 9 underwent EX-PRESS implantation. The mean IOP and number of medications used at the last follow-up decreased significantly compared with that before surgery (*P* < 0.001). The total success rate for all eyes including complete success (7/23) and qualified success (7/23) was 60.9% (14/23) at 1 year. The total success rate in the AGV group was 78.6% (11/14), whereas it was 33.3% (3/9) in the EX-PRESS group; the difference between the 2 groups was significant (*P* < 0.05).

**Conclusion:**

For glaucoma secondary to SO emulsification, glaucoma implants could be effective at lowering IOP, and AGVs might produce better outcomes than EX-PRESS devices.

## 1. Introduction

Silicone oil (SO) is widely used in the management of complicated retinal detachment. However, a series of complications can be caused by intraocular SO tamponade, including transient or permanent intraocular pressure (IOP) elevation [1–3]. A major reason for the occurrence of glaucoma secondary to SO tamponade is SO emulsification. The incidence of open-angle glaucoma (OAG) after SO emulsification varies from 11% to 56% [4, 5]. The underlying mechanism might be the migration of SO droplets into the anterior chamber, which could directly obstruct the trabecular meshwork or cause inflammation in it [6]. In addition, long-term contact between the emulsified SO and the trabecular meshwork may result in sclerosis and collapse of the trabecular meshwork. Silicone-laden macrophages have been found within the trabecular meshwork of pathologic specimens, suggesting their role in the obstruction of the outflow tract [7]. Medical control of IOP is the first choice treatment. Budenz et al. [8] reported a success rate of 69% for medical IOP control at 6 months, which dropped to 48% at 24 months. If IOP cannot be satisfactorily controlled, silicone removal is recommended. Additional invasive procedures include trabeculectomy, cycloablation, and implantation of glaucoma drainage devices.

Considering that trabeculectomy has a poor prognosis due to conjunctival scarring from previous vitreoretinal surgery, glaucoma drainage devices could be an alternative treatment approach. Widely used glaucoma drainage devices include the EX-PRESS glaucoma filtration device (Alcon Laboratories, Inc., Nevellan, Israel) and the Ahmed glaucoma valve (AGV) (New World Medical, Inc., Rancho Cucamonga, CA, USA). Errico et al. [9] reported an overall success rate of 73% with the EX-PRESS implanted in patients who were diagnosed with glaucoma with SO emulsification in a 24-month follow-up period. Reports of AGV use for glaucoma with SO emulsification are limited. Ishida et al. [10] reported successful control of IOP in 47% of patients with SO endotamponade over 4 years.

In the current study, we retrospectively reviewed a series of patients who were diagnosed with glaucoma secondary to SO emulsification and received EX-PRESS or AGV implants. We recorded their ocular data and evaluated the surgery results for OAG secondary to SO emulsification.

## 2. Methods

### 2.1. Participants

**Figure 1:**
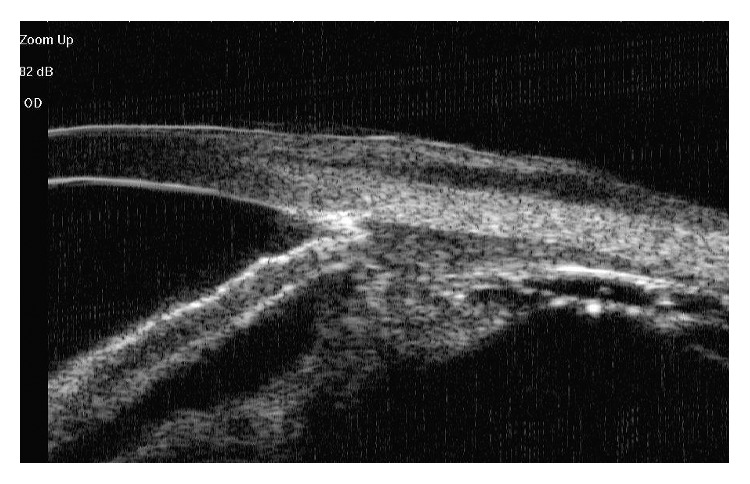
Image of UBM of a patient with glaucoma after PPV and SO injection showed an open chamber angle and several dotted high-echoes in the angle suggesting the SO emulsification.

This was a retrospective case-control study. A total of 23 eyes from 23 patients who were diagnosed with OAG secondary to SO emulsification at the Department of Ophthalmology, Eye, Ear, Nose and Throat Hospital, affiliated with Shanghai Fudan University, from January 2012 to June 2015, were included in the study. Patients with high IOP before pars plana vitrectomy (PPV) or whose IOP elevation was thought to be caused by other factors, including synechial angle closure, rubeosis iridis, SO overfilling, pupillary block, inflammation, or steroid-induced glaucoma, were excluded from the study. Not only eyes with rhegmatogenous retinal detachment (RRD) but also cases with ocular trauma, foreign body, or endophthalmitis were included. The previous surgery was PPV and SO injection in all of the eyes. SO with a viscosity of 5,000 centistokes was used. The diagnosis of glaucoma secondary to SO emulsification was confirmed by the presence of SO emulsion droplets in the anterior chamber and high IOP. The appearance of SO microglobules in the chamber angle was determined via a gonioscope and an ultrasound biomicroscope (Figure 1). In all of the eyes, SO removal was performed and IOP-reducing medication (≥2 types) was administered but was ineffective. Data on the general characteristics of included patients were compared between the AGV and EX-PRESS groups. Detailed ophthalmological examinations were performed 1 day before antiglaucoma surgery, including best-corrected VA, Goldmann applanation tonometry, slit-lamp examination, and fundus examination. VA was measured via a decimal chart. Previous ocular history and the use of topical or general antiglaucoma medications were recorded. In our study, all of the participants signed informed consent forms. The study was approved by the institutional review board of the Eye, Ear, Nose and Throat Hospital of Fudan University and was performed in accordance with the tenets of the Declaration of Helsinki.

### 2.2. Surgical Techniques and Measurements

Both the EX-PRESS P200 implant (Alcon Laboratories) and the AGV implant model FP7 (New World Medical, Inc.) were used in all 23 eyes. All surgeries were performed under peribulbar anesthesia by the same surgeon.

For EX-PRESS shunt implantation, a limbal-based conjunctival dissection was created after a sub-Tenon injection of 1% lidocaine. A 4.0 mm × 3.5 mm partial thickness trapezoidal scleral flap was created, and a sponge soaked with 0.4 mg/mL of MMC was applied under the conjunctival and scleral flaps for 3 min followed by rinsing with 20 mL of 0.9% sodium chloride. A 26-gauge needle was then used to make a microincision under the scleral flap in the center of the blue-gray transition zone, and the EX-PRESS drainage device was inserted into the anterior chamber. The scleral flap and the conjunctiva were then closed as in a trabeculectomy.

In the AGV group, a conjunctival incision was made at the limbus, and 0.4 mg/mL of MMC was applied under the conjunctival flap for 3 min and then washed out. The AGV was inserted through the conjunctiva and Tenon's capsule and sutured to the sclera approximately 8 mm behind the limbus. The tube was trimmed to an appropriate length and inserted into the anterior chamber with the bevel facing anteriorly through a scleral track 2 mm behind the limbus created using a 23-gauge needle. A rectangular donor scleral patch graft was created and then fixed over the exposed part of the tube using 10-0 nylon sutures. The conjunctiva and Tenon's capsule were repaired using absorbable sutures.

The postoperative follow-up visits were scheduled at 1 day, 1 month, 3 months, 6 months, and 12 months after surgery. Ocular parameters, including VA and IOP, and the number of medications used were recorded at each visit. Three outcomes were defined for this study: eyes with normal IOP (<21 mmHg) at the 12-month follow-up were considered successes, and among these, those controlled by glaucoma medications (≤3) or needle revision were considered qualified successes and those not requiring any medication or needle revision were regarded as complete successes. Eyes with high IOP that could not be controlled with medication and that underwent other surgical intervention, such as surgical scar removal and cyclocryotherapy, were considered failures and were removed from the follow-up sample. In eyes classified as failures, IOP, medication, and VA data were collected beyond the failure date until the last available follow-up. Data on the IOP and medications at each visit were compared between the AGV and EX-PRESS groups.

### 2.3. Statistical Analysis

The independent sample *t*-test and Mann–Whitney *U* test (SPSS, Inc., Chicago, IL, USA) were used for the comparison of data between the two groups depending on whether the data followed a normal distribution. The K–S test was used to determine the normality of data. Kaplan–Meier survival analysis was performed for the entire group and for each type of surgical procedure. Categorical variables were compared using the log-rank test, taking into account the time of failure and time of loss to follow-up for each subject. *P* < 0.05 was considered statistically significant.

## 3. Results

A total of 23 eyes from 23 patients (18 males and 5 females) with a mean age of 46.0 ± 14.8 years (range 14–75 years) were included in this study. The surgical indications of PPV were RRD in 18 eyes and ocular injury or consequent ocular infection in 5 eyes. Among the eyes with RRD, fundus manifestations were giant retinal hole, multiple retinal holes, high myopia with macular holes, choroidal detachment, and severe proliferative vitreoretinopathy (PVR) after PPV failure. Ocular injuries included intraocular foreign bodies caused by penetrating injuries and ocular rupture. Two eyes were diagnosed with traumatic endophthalmitis. The mean silicone tamponade duration was 10.8 ± 10.4 months (range 3–46 months). Ten eyes underwent extended tamponade due to a high risk of retinal redetachment. The other 13 eyes were lost to follow-up after PPV surgery until the patients felt eye pain and were diagnosed with high IOP. All of the eyes underwent surgery for SO removal with good retinal adhesion. The mean duration between SO removal and antiglaucoma surgery was 3.1 ± 1.6 months (range 1–6 months). There were no significant differences in the data between the AGV and EX-PRESS groups. The characteristics of the AGV and EX-PRESS groups are shown in Table 1. The mean preoperative IOP was 34.3 ± 7.6 mmHg (range 25–49 mmHg), and visual acuity (VA) ranged from “hand motion” to 0.25. The mean number of topical antiglaucoma drops used before surgery was 3.4 ± 0.9.

**Figure 2:**
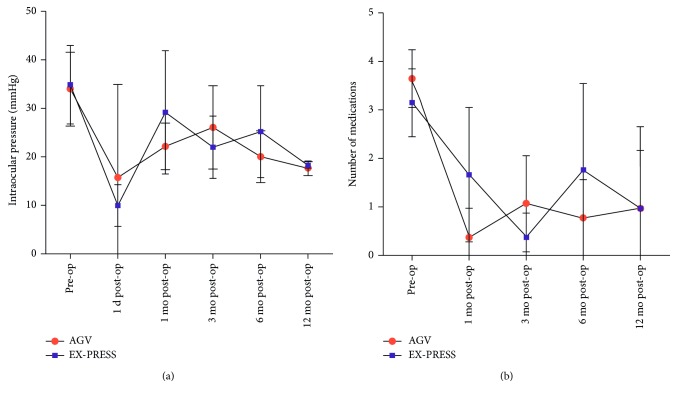
(a) The line graph of intraocular pressure before surgery and one day, one month, three months, six months, and twelve months after surgery. (b) The line graph of the number of medications used before surgery and one day, one month, three months, six months, and twelve months after surgery.

**Figure 3:**
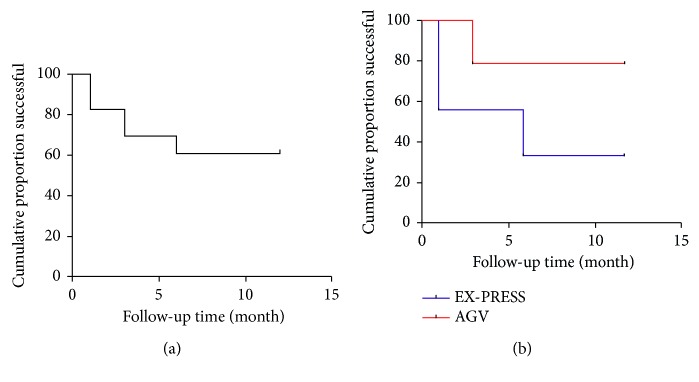
(a) The Kaplan–Meier survival curve for all of the eyes (all data referred to total success rate). (b) The Kaplan–Meier survival curve based on surgical procedures (Ahmed glaucoma valves or EX-PRESS) (all data referred to total success rate).

Among the 23 eyes, 14 underwent AGV implantation and 9 underwent EX-PRESS shunt implantation. After a mean follow-up of 8.4 ± 4.8 months, IOP and the number of antiglaucoma medications decreased (Figure 2). In the AGV group, the mean IOP was 15.8 ± 9.2 mmHg the day after surgery and 17.7 ± 1.5 mmHg 12 months after surgery, and in the EX-PRESS group, the corresponding values were 10.0 ± 4.3 mmHg and 18.3 ± 0.6 mmHg, respectively. The mean numbers of glaucoma medications in use at the 12-month visit were 1.0 ± 1.2 in the AGV group and 1.0 ± 1.7 in the EX-PRESS group. There were no significant differences in IOP and the number of medications between the AGV and EX-PRESS groups at each visit except in the number of medications at the 3-month visit (*P*=0.033).  Table 2 shows a summary of IOPs and the numbers of glaucoma medications in the AGV and EX-PRESS groups before surgery and at each follow-up time-point thereafter and the statistical results. At 12 months, 14 of the 23 eyes (60.9%) were considered a success, of which 7 (30.4%) were a complete success and 7 (30.4%) were a qualified success. Nine eyes still exhibited high IOP after the administration of more than 3 antiglaucoma medications and were judged as failures. At 1, 3, 6, and 12 months after surgery, the respective cumulative success rates were 100%, 82.6%, 69.6%, and 60.9%. At the end of follow-up, the success rates were 78.6% in the AGV group and 33.3% in the EX-PRESS group, and this difference was statistically significant (*P*=0.022).  Figure 3 shows the Kaplan–Meier survival curves for all eyes based on surgical procedures. The main reason for surgical failure was thought to be scar formation. Two eyes in the AGV group were judged as failure after 3-month follow-up. In the EX-PRESS group, four eyes and 2 eyes were judged as failure after 1-month and 6-month follow-up, respectively. The conjunctiva was reopened, and fibrous scarring around the filtering bleb or Tenon cyst encapsulating the valve body was removed in 8 patients. Another eye with uncontrollable high IOP had no light perception at the 6-month follow-up, and cyclocryotherapy treatment was administered to relieve the ocular pain. The distribution of all failure cases in the study is presented in Table 3.

After surgery, 11 eyes underwent needling revision with 5-fluorouracil within 1 to 3 months. Complications encountered in our study included AGV tube obstruction by inflammatory exudation and consequent high IOP (up to 42 mmHg) in 1 eye, which was treated using a yttrium aluminum garnet laser and transient hypotony soon after surgery, which resolved spontaneously in 2 eyes in the EX-PRESS group. No corneal decompensation or endophthalmitis was encountered.

## 4. Discussion

SO is the favored material for tamponade in eyes with PVR or giant retinal tears [11]. Elevation of IOP is a common complication of PPV with SO injection. Emulsification of the SO has been reported to be associated with a rise in IOP in the late period [12], the associated factors of which are mentioned in Introduction. In our study, two different glaucoma drainage implants, the EX-PRESS P200 and the AGV model FP7, were used as alternative surgery options to treat glaucoma secondary to SO emulsification. The success rate over a 12-month period in the AGV model FP7 group was 78.6%, and that in the EX-PRESS P200 group was 33.3%. The results were statistically significant (log-rank test, *P* < 0.05). The AGV model FP7 showed a likely better clinical outcome than the EX-PRESS P200.

SO emulsification is a multifactorial process in which the biochemical properties of SO, endotamponade duration and ocular inflammation all play roles. It has been reported that 1000 centistokes of oil is more likely to cause elevated IOP than 5000 centistokes of oil [13]. A previous study reported that SO emulsification is independent of the duration of SO endotamponade [14]. In our opinion, a long SO tamponade duration might lead to the elevation of IOP. In our study, the SO endotamponade ranged from 3 months to 46 months with a mean duration of 10.8 months, which was longer than a normal SO tamponade period of 3–6 months.

SO removal is performed to reduce IOP when there is a low risk of retinal redetachment. Budenz et al. [8] reported a success rate of 64% in patients who underwent SO removal alone in a study involving a 36-month follow-up period. At the stage when SO removal alone plays no role, trabeculectomy—a conventional filtration surgery—can be used. However, this approach produces limited results in the management of glaucoma secondary to SO emulsification. Conjunctival scarring from previous PPV surgery always results in a poor prognosis [6]. Gedde et al. [15] reported a multicenter randomized clinical trial in which patients with previous ocular surgery underwent a tube shunt placement or a trabeculectomy, and in that trial, the tube shunt yielded a higher success rate than trabeculectomy with mitomycin C (MMC) over a 5-year follow-up period.

In our EX-PRESS group, the surgical success rate was 33.3%, which is much lower than the 80% success rate reported by Mendoza-Mendieta et al. [16] for eyes with primary OAG. In our opinion, this major difference could be due to the previous ocular surgery in the cases in our study. AGV is a widely used implant in refractory glaucoma. Ishida et al. [10] reported a success rate of 80% with AGV implants in eyes with SO endotamponade, which is similar to the rate of 78.6% observed in our study. In all cases included in our study, the anterior conjunctiva was opened in previous interventions, which rendered the formation of subconjunctival scarring inevitable. Given that the main reasons for the failure of implant surgery are reportedly bleb encapsulation and the proliferation of subconjunctival fibrous tissue [17], we considered the previous conjunctival scarring of each patient when choosing the type of implant. We preferred to use the AGV in patients with more serious anterior conjunctival scarring because EX-PRESS implants are placed just behind the corneal limbus, whereas AGV implants are placed 8–10 mm behind the limbus, where previous surgical disturbances were fewer. Although we considered these factors, we still observed a better result in the AGV group at the 12-month follow-up time-point, which strongly supports the use of AGV in eyes with a history of previous ocular surgery. Success rate Kaplan–Meier survival curves also indicated that there was minimal change in scar formation in the AGV group or the EX-PRESS group between 6 and 12 months.

We applied bleb needling with 5-fluorouracil in 11 eyes with IOP elevation during the follow-up period, and IOP was lowered effectively in 6 of these eyes. Needling seemed most likely to succeed in eyes with IOP lower than 30 mmHg, possibly because in these eyes, fibrosis formation was not particularly severe and needling together with antimetabolite treatment could therefore be effective. The relationship between patient age and the success rate of needling reported by Quaranta et al. [17] was not apparent in our study, possibly because the number of patients who received needling in our study was limited.

During the surgical process, we identified an additional advantage of AGV implantation compared to EX-PRESS implantation. In our study, all of the eyes had previously undergone PPV, and aqueous fluid had filled the vitreous cavity. The straightforward aqueous outflow procedure adopted in the EX-PRESS group caused large IOP fluctuations during surgery and hypotony soon after surgery. Conversely, the AGV is a restrictive valve device designed to prevent hypotony during and after surgery, which renders the surgical process safer [18].

In our study, although VA was recorded at each follow-up visit, these data were not used in the statistical analysis because all of the eyes had previous retinal disease and preoperative vision was poor in most of them (ranging from “hand motion” to “counting fingers”). We believe that the change in VA during the follow-up period might not be completely attributable to IOP fluctuation.

The limitations of this study include the small sample sizes of both groups, which may challenge the validity of the survival analysis in our study, and the retrospective nature of the study. Given that IOP can be reduced and stabilized to a large extent after the extraction of silicone oil in conjunction with the administration of one or two medicines, glaucoma surgery is only required in relatively few cases. In the past 4 years, as the largest eye institute in the east of China, we have encountered 23 cases based on strict exclusion criteria. More prospective studies with larger cohorts of subjects are needed to support the present findings.

## 5. Conclusions

In summary, in this retrospective study, glaucoma implants were effective in lowering IOP for eyes with glaucoma secondary to SO emulsification, and AGVs might produce better outcomes than EX-PRESS devices. These results might provide some insight for clinical practice.

## Figures and Tables

**Table 1:** Baseline characteristics of the study patients.

Characteristic	AGV	EX-PRESS	*P* value
	Patients (*n*=14)	Patients (*n*=9)	
Age (years), mean ± SD (range)	46.8 ± 13.5 (15–65)	44.3 ± 17.4 (14–75)	0.708
*Gender,n* (%)			
Male	11 (78.6)	7 (77.8)	
Female	3 (21.4)	2 (22.2)	
*Indication for PPV,n* (%)			
RRD	11 (78.6)	7 (77.8)	
Giant retinal hole or multiple retinal holes	4 (28.6)	3 (33.3)	
Macular hole caused by high myopia	2 (14.3)	2 (22.2)	
Associated with choroidal detachment	2 (14.3)	1 (11.1)	
PVR after failed PPV	2 (14.3)	2 (22.2)	
Ocular injury	3 (21.4)	2 (22.2)	
Penetrating injury	3 (17.4)	1 (11.1)	
With endophthalmitis	1 (8.7)	1 (11.1)	
With IOFB	2 (14.3)	0 (0)	
Rupture	0 (0)	1 (11.1)	
Tamponade duration (months), mean ± SD (range)	11.3 ± 8.2 (3–31)	10.0 ± 13.7 (3–46)	0.153
Duration between removal of silicone oil and antiglaucoma surgery (months), mean ± SD (range)	2.8 ± 1.6 (1–5)	3.6 ± 1.6 (1–6)	0.240
Axial length of the eye (mm), mean ± SD (range)	25.5 ± 2.4 (23.3–30.7)	26.9 ± 3.0 (24.2–31.1)	0.208
Anterior chamber depth (mm), mean ± SD (range)	3.1 ± 0.5 (2.3–3.8)	3.0 ± 0.5 (2.1–3.5)	0.801

AGV: Ahmed glaucoma valve; EX-PRESS: EX-PRESS glaucoma filtration device; SD: standard deviation; PPV: pars plana vitrectomy; RRD: rhegmatogenous retinal detachment; PVR: proliferative vitreoretinopathy; IOFB: intraocular foreign body.

**Table 2:** Preoperative and follow-up outcomes.

		Preoperative	1 day	1 month	3 months	6 months	12 months	*P*1 value
AGV	Number of patients	14	14	14	14	11	11	—
	IOP	34.0 ± 7.6	15.8 ± 9.2	22.2 ± 4.8	26.1 ± 8.6	20.1 ± 5.3	17.7 ± 1.5	0.000
		(25–49)	(7.9–42)	(15–30)	(16–42)	(9.1–30)	(15–20)	—
	Medications	3.7 ± 0.6	0	0.4 ± 0.6	1.1 ± 1.0	0.8 ± 0.8	1.0 ± 1.2	0.000
		(3–5)		(0–2)	(0–3)	(0–2)	(0–3)	—

EX-PRESS	Number of patients	9	9	9	5	5	3	—
	IOP	34.9 ± 8.1	10.0 ± 4.3	29.2 ± 12.7	22.0 ± 6.4	25.2 ± 9.4	18.3 ± 0.6	0.000
		(27–49)	(5.4–17)	(8–41)	(17–30)	(17–39)	(18–19)	—
	Medications	3.2 ± 0.7	0	1.7 ± 1.4	0.4 ± 0.5	1.8 ± 1.8	1.0 ± 1.7	0.000
		(2–4)		(0–3)	(0–1)	(0–4)	(0–3)	—

*P*2 value	IOP	0.792	0.051	0.195	0.485	0.307	0.524	—
	Medications	0.100	—	0.033	0.138	0.314	0.801	—

AGV: Ahmed glaucoma valve; EX-PRESS: EX-PRESS glaucoma filtration device; IOP: intraocular pressure. The values shown are means ± standard deviation, with the range in brackets. *P*1 value: comparison between preoperative and 12 months. *P*2 value: comparison between AGV group and EX-PRESS group at each time-point.

**Table 3:** Distribution of surgical failures in this study.

	EX-PRESS	AGV	Treatment
1 month postoperative	4	0	Surgical scar removal
3 months postoperative	0	3	Surgical scar removal
6 months postoperative	2	0	Surgical scar removal/cyclocryotherapy

AGV: Ahmed glaucoma valve; EX-PRESS: EX-PRESS glaucoma filtration device.

## Data Availability

The data used to support the findings of this study are available from the corresponding author upon request.
